# Antimicrobial Susceptibility and Molecular Identification of Antibiotic Resistance Enteric Bacteria Isolated From Pigeon Feces in the City of Jeddah, Saudi Arabia

**DOI:** 10.7759/cureus.67371

**Published:** 2024-08-21

**Authors:** Mohamed Elmutasim A Elsheikh, Maher Alandiyjany, Manal El Said, Faten Abouelmagd, Nadeem Ikram, Muhammad Awais, Elshiekh B Khidir, Wafaa M Abdulrahaman, Hassan Elsiddig Hag Elsafi, Omeima Salih

**Affiliations:** 1 Department of Microbiology, General Medicine Practice, Batterjee Medical College, Jeddah, SAU; 2 General Medicine Practice, Batterjee Medical College, Jeddah, SAU; 3 Department of Clinical Laboratory Sciences, Faculty of Applied Medical Sciences, Umm Al-Qura University, Makkah, SAU; 4 Department of Microbiology and Infection Prevention and Control Unit, Theodor Bilharz Research Institute, Giza, EGY; 5 Department of Medical Parasitology, Faculty of Medicine, Sohag University, Sohag, EGY; 6 Department Microbiology, Ahfad Centre for Science and Technology, Ahfad University for Women, Omdurman, SDN; 7 Department of Public Health, School of Health Sciences, Ahfad University for Women, Omdurman, SDN

**Keywords:** saudi arabia, jeddah, args, pigeon, enteric-bacteria

## Abstract

Background

Due to their potential to carry a wide range of bacteria, pigeon feces may contribute to the spreading of infectious diseases in urban settings.

Objective

This study analyzed the presence of enteric bacteria from pigeon feces in Jeddah and their antimicrobial susceptibility and described the molecular characteristics of the carbapenem resistance genes it produced.

Method

Two hundred twenty-five pigeon feces specimens were collected from eight parks in Jeddah. Conventional microbiology techniques were employed to identify the isolated bacteria, and the automated Vitek2® system (bioMérieux, Marcy-l'Étoile, Lyon, France) provided additional confirmation. Kirby-Bauer disk diffusion method was utilized to screen for antimicrobial resistance. Only 50 antibiotic-resistance isolates further underwent molecular diagnosis for testing groups of carbapenems-encoding genes (blaNDM, blaSIM, and blaAIM), using multiplex polymerase chain reaction (PCR).

Result

Of the 50 antibiotic-resistant isolates, 28% (14/50) were *Klebsiella pneumoniae*, 24% (12/50) were *Enterobacter cloacae*, and 48% (24/50) were *Escherichia coli*. Ninety percent (90%) of the isolates showed resistance to cefuroxime, 56% to gentamicin, 52% to amoxicillin/clavulanic acid, and 100% to meropenem. NDM beta-lactamase was the most often discovered gene (26%) and was followed by AIM beta-lactamase (5%)

Conclusion

According to this study, there may be a chance for resistant *K. pneumoniae, E. cloacae*, and* E. coli *to spread amongst several hosts within the same area. Consequently, to prevent the continued occurrence and dissemination of resistant strains among other hosts in the same location, it is essential to monitor the AMR (antimicrobial resistance) of *E. coli, E. cloacae*, and *K. pneumoniae* from pigeons.

## Introduction

There are numerous pigeons throughout the world, in both countryside and cities, interacting closely with people and other animals [1-3]. Pigeon feces may contribute to the spread of infectious illnesses in the environment because they may contain various bacteria [1,4]. These could include resilient human pathogens such as strains of diarrheagenic Escherichia coli, creating the potential for exposure and human infection [1,2,5,6]*.*

Humans and other animals have been documented to develop digestive disorders because of diarrheagenic *E. coli *strains [2,7]. However, animals may also be a source of antimicrobial resistance determinants, which can then spread to humans [3,8,9]*.* It is commonly recognized that commensal and pathogenic strains of *E. coli* can have several genetic markers associated with microbial resistance and exhibit varying rates of resistance [1,10]. 

The emergence of antibiotic resistance in Gram-negative bacteria presents a significant risk to human and animal treatment, both clinically and financially. Environmental species include *Pseudomonas, Stenotrophomonas, Aeromonas*, and Enterobacteriaceae spp. are multi-resistant opportunistic pathogens linked to devastating illnesses in both humans and animals. The widespread Enterobacteriaceae family of Gram-negative bacteria often colonizes the gastrointestinal environment. They are a major cause of contamination in food and water and one of the most important opportunistic pathogenic organisms in the therapeutic setting. They are connected to numerous hospital- and community-acquired diseases, and they can interchange genetic data via horizontal gene transfer in addition to acquiring antibiotic resistance genes (ARGs) [8-10]. 

Antibiotic molecules can be found outside of medical facilities in soil and water sources thus they are created by ambient microbes or eliminated by people and animals [11]. Furthermore, it is well known that co-selection by heavy metals utilized in animal husbandry and aquaculture encourages the emergence of antibiotic resistance in bacteria [12]. Several studies have demonstrated that MDR (multidrug resistance) bacteria can be found in natural environments as reservoirs and that the environmental metagenome, often known as the environmental "resistome," is home to a wide variety of ARGs that can spread horizontally to new hosts [13].

The most well-known property of carbapenemases, which are β-lactamases that inactivate carbapenems and the majority of β-lactam antibiotics, is that they can impart resistance to β-lactams. These include metallo-β-lactamases, such as the SIM (Seoul imipenemase), NDM (New Delhi metalo-beta-lactamase), AIM (Adelaide imipenemase), and VIM (Verona integron-encoded metallo-β-lactamase) enzyme families, which Gram-negative bacteria have gained through transferable elements, and serine carbapenemases, such as the widely distributed KPC (*Klebsiella pneumoniae* carbapenemase) family of enzymes [13].

Serious health issues are raised by the environmental co-contamination that occurs with the rise of MDR bacteria in humans, animals, and birds [14,15]. Wild birds can act as reservoirs for coliform bacteria that carry antibiotic-resistance genes, such as *E. coli*. These birds have yielded several hazardous kinds of bacteria that have been isolated. Water contact and food acquisition are the ways that resistant bacteria spread from people or veterinary sources to wild animals [16,17]. 

Drug resistance in infected pigeons in Jeddah, western Saudi Arabia is not known. The effects of antibiotic sensitivity testing from potential pathogenic bacteria isolated from pigeons on human health, even when considering different ecological habitats of the world, are unknown. To assess the likelihood that these pigeons are home to possible human intestinal drug-resistant pathogens, this study analyzes the presence of enteric bacteria isolated from pigeons' feces in Jeddah and their antimicrobial sensitivity patterns and describes the molecular characteristics of the carbapenems resistance genes it produced. 

## Materials and methods

Study design 

This was a cross-sectional, descriptive, laboratory-based study.

Sampling

Using sterile cotton swabs, 225 pigeon feces samples were collected from different sites in eight parks of the city of Jeddah, Saudi Arabia, between May 2021 and March 2022. 

The inclusion criteria were isolated Gram-negative rods bacteria, while the exclusion criteria were non-glucose fermenter and oxidase-positive Gram-negative rods bacterial isolates.

Microbiological identification

All swab samples were enriched in buffered peptone water at 37 °C for 24 h. Subsequently, they were sub-cultured on MacConkey agar and incubated at 37 °C overnight. The plates were examined for lactose fermenting or non-lactose fermenting bacteria. Then purified isolates were morphologically characterized by the Gram stain method. Presumptive identification was done using biochemical tests including oxidase, indole, citrate, catalase, urea hydrolysis, and sugar fermentation, and then confirmed by Vitek2 automated system (bioMérieux, Marcy-l'Étoile, Lyon, France). 

Antimicrobial susceptibility test

All bacteria isolates were investigated to antibiogram against eight commonly used antibiotics, i.e., meropenem (10 µg)(carbapenem), ceftazidime (30 µg) (third-generation cephalosporin), cefuroxime (30 µg) (second-generation cephalosporin), cefotaxime(30µg) (third-generation cephalosporin), trimethoprim/sulfamethoxazole (1.25/23.75 µg)( dihydrofolate reductase inhibitor/sulfonamide), amoxicillin + clavulanic acid (25/10 µg) (penicillin-like antibiotics/beta-lactamase inhibitors), ciprofloxacin (5 µg) (fluoroquinolone), and gentamicin (10 µg) (aminoglycosides ), utilizing the disk diffusion techniques developed by Bauer et al. [18]. To determine whether the isolates were resistant or sensitive to the tested antimicrobial agents, the zone of growth inhibition was lastly compared to standards given by the Clinical and Laboratory Standards Institute [19]. Resistance to three or more antimicrobial groups is considered multidrug resistance or MDR. Only 50 antibiotic-resistance isolates further underwent molecular diagnosis. 

DNA extraction

Following the manufacturer's instructions, 50 genomic DNA samples were extracted from resistant bacteria using the QIAamp DNA Microbiome Kit® (QIAGEN, Valencia, CA, USA). 

Multiplex PCR

Multiplex PCR was done by DreamTaq Green PCR Master Mix (2X) (cat# K1081) (Thermo Fisher Scientific Inc., Waltham, USA) using the following primers (Table 1): 

**Table 1:** Primers used in this study a= Primers (F) forward and (R) reverse, b=sequence from 5 ends to 3 ends, c=beta lactamase genes, d=length or size of the genes in (bp) base pair unit, NDM=New Delhi metalo-beta-lactamase, AIM=Adelaide imipenemase, SIM=Seoul imipenemase

(a) Primer	(b) Sequence (5′–3′)	(c) Gene Product	(d) amplicon size (bp)
AIM-F	CTGAAGGTGTACGGAAACAC	blaAIM	322
AIM-R	GTTCGGCCACCTCGAATTG		
SIM-F	TACAAGGGATTCGGCATCG	blaSIM	570
SIM-R	TAATGGCCTGTTCCCATGTG		
NDM-F	GGTTTGGCGATCTGGTTTTC	blaNDM	621
NDM-R	CGGAATGGCTCATCACGATC		

The PCR protocol was performed according to manufacturer instructions with a total volume of 20 ul (Table 2). The PCR amplification for DNA samples was performed using Verti™ Thermal Cyclers (Applied Biosystems, Waltham, USA) under the following thermal cycling conditions: 10 min at 94°C and 36 cycles of amplification consisting of 30 s at 94°C (denaturation), 40 s at 52°C (annealing), and 50 s at 72°C, with 5 min at 72°C for the final extension [20]. 

**Table 2:** PCR Protocol PCR=polymerase chain reaction

	Reagent	Volume /Sample
1	2X Master Mix	10 µl
2	10 μM Primers (F & R) for AIM	2.0 µl
3	10 μM Primers (F & R) for SIM	2.0 µl
4	10 μM Primers (F & R) for NDM	2.0 µl
5	DNA (50 ng / µl )	2.0 µl
6	Nuclease Free Water	2.0 µl
	Total Volume	20 µl

Gel electrophoresis

The size and quality of the PCR amplification products were confirmed using agarose gel electrophoresis. 2% gel stained with Cybersafe DNA dye (Invitrogen, Carlsbad, USA) after being prepared with Ultrapure Agarose(Cleaver Scientific,** **Thistle, Rugby, UK)** **and 1XTBE buffer. Each gel well was filled with 4 μl of the PCR product. The samples were run alongside a DNA ladder (100-1000) (GeneON, Ludwigshafen, Germany) that was run for 30 minutes at 100 mV. DNA fragments were visible with the Gel Doc system imager system UV transilluminator (Bio-Rad Laboratories, Hercules, USA). 

Statistical analysis 

Data were analyzed using multiple correspondence analysis (MCA) and the Chi-square test was used to obtain p-values to identify significant associations between each two variables using IBM SPSS software version 26 (IBM Corp., Armonk, USA). Statistics were deemed significant if p<0.05. 

Ethics statement 

The sites, activities, and field investigations that were detailed did not require any specific licenses. The field experiments did not involve endangered or protected animals, the sampled sites were not privately owned, and the researchers had no prior physical interaction with pigeons. 

## Results

Isolated bacteria

**Figure 1:**
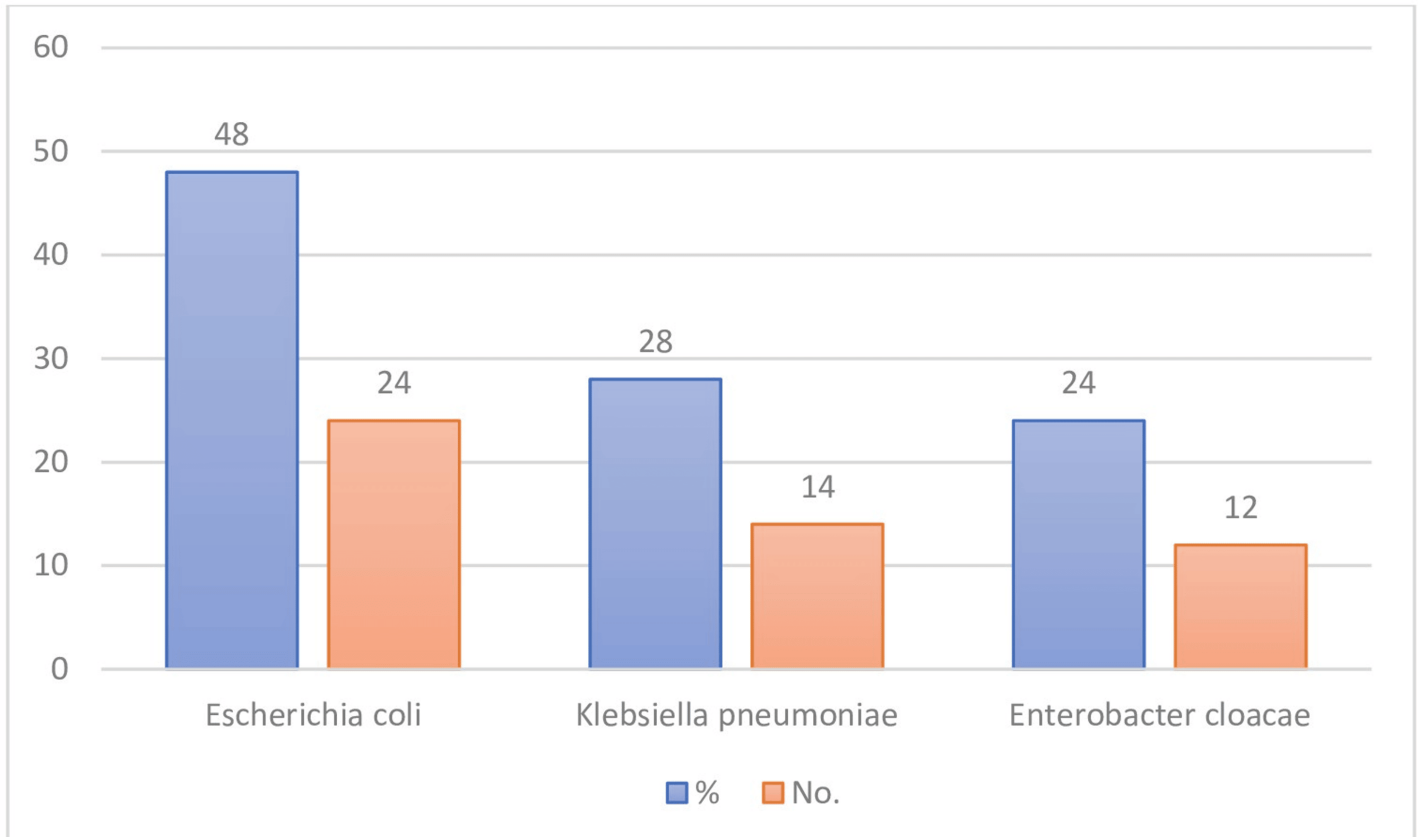
Percentage and numbers of bacterial isolates from 50 pigeon fecal samples

Among 225 pigeon feces samples collected, only 22% (50/225) of isolates were confirmed antibiotic-resistant Enterobacteriaceae. The 50 antibiotic-resistance isolates were identified by biochemical tests and were confirmed by the Vitek2 automated system (Bio Merieux). Among these 50 isolates, 48% (24/50) were *E. coli*, 28% (14/50) were *K. pneumoniae*, and 24% (12/50) were *E. cloacae* (Figure 1). 

Antibiotic resistance

Among 50 species of *E. coli, E. cloacae, and K. pneumoniae*, not a single isolate was susceptible to all antibiotics, however, all isolates were meropenem-resistant (100%). 45 isolates (90%) were cefuroxime-resistant, 28 (56%) isolates were gentamicin-resistant, and 26 isolates (52%) were amoxicillin/clavulanic acid-resistant (Table 3). 

Seventeen (34%) isolates were not susceptible to one or two antibiotics. Additionally, multidrug resistance was recognized in 66% of isolates that included more than three antibiotic groups. In contrast, very few bacteria showed resistance to ceftazidime, cefotaxime, and ciprofloxacin in the studied bacterial species. The susceptibility and resistance characteristics of the tested isolates are summed up in Table 3. Regarding beta-lactams, the findings showed that the highest resistance rate was observed for carbapenems. Moderate levels were observed compared to other beta-lactams such as first and second-generation cephalosporins. The lowest resistance rates were observed for third-generation cephalosporins and fluoroquinolones (Table 3). 

**Table 3:** Susceptibility pattern on pigeon faces Bacterial isolates in Jeddah, KSA The proportion comparison χ² test performed for a value of α=5%; * significant value MEM=meropenem, CAZ=ceftazidime, CXM=cefuroxime, CTX=cefotaxime, SXT=triméthoprim+sulfamethoxazole, CIP=ciprofloxacin, AMC=amoxicillin+clavulanic acid, CN= gentamicin

Isolated bacteria	No	Susceptibility	MEM	CAZ	CXM	CTX	SXT	CIP	AMC	CN
Escherichia coli	24	Frequency of susceptibility	0	6	0	5	19	20	8	11
		Frequency of intermediate	0	16	2	17	1	1	5	0
		Frequency of resistance	24	2	22	2	4	3	11	13
Enterobacter cloacae	12	Frequency of susceptibility	0	2	0	3	4	12	2	2
		Frequency of intermediate	0	10	0	9	0	0	0	0
		Frequency of resistance	12	0	12	0	8	0	10	10
Klebsiella pneumoniae	14	Frequency of susceptibility	0	7	0	10	10	12	5	9
		Frequency of intermediate	0	7	3	4	3	2	4	0
		Frequency of resistance	14	0	11	0	1	0	5	5
P. value			-	0.197	0.179	0.009*	0.001*	0.201	0.124	0.05*

There were statistically significant differences in cefotaxime sensitivity and resistance pattern for the three species of isolated bacteria as *K. pneumoniae* was found sensitive (P=.009) compared to *E. coli and E. cloacae *(resistant), another statistically significant difference was observed with triméthoprim+sulfamethoxazole ( P=.001) and gentamicin (P=.05) as *K. pneumoniae *and* E. coli* were sensitive while *E. cloacae *were found to be resistant (table 3). 

Genotypic analysis

**Figure 2:**
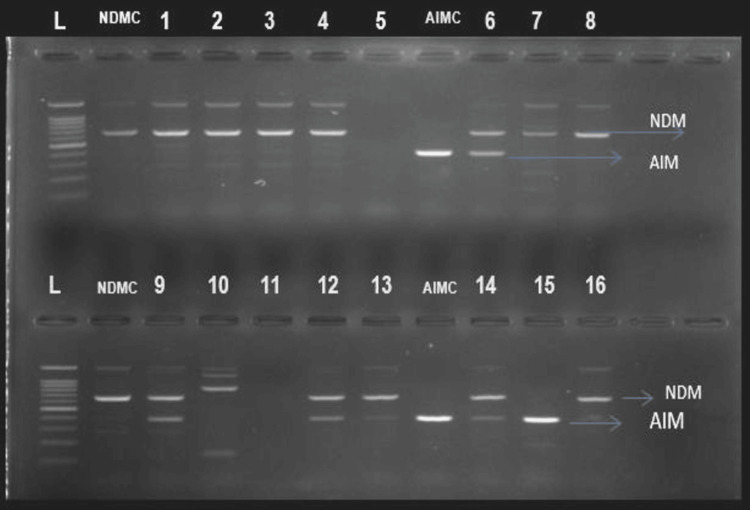
2% gel electrophoresis PCR results using AIM, SIM, and NDM genes for bacteria isolated from pigeon faces Ladder 100 bp, NDMC=NDM positive control, AIMC=AIM positive control, PCR=polymerase chain reaction, NDM=New Delhi metalo beta-lactamase, AIM=Adelaide imipenemase, SIM=Seoul imipenemase

**Figure 3:**
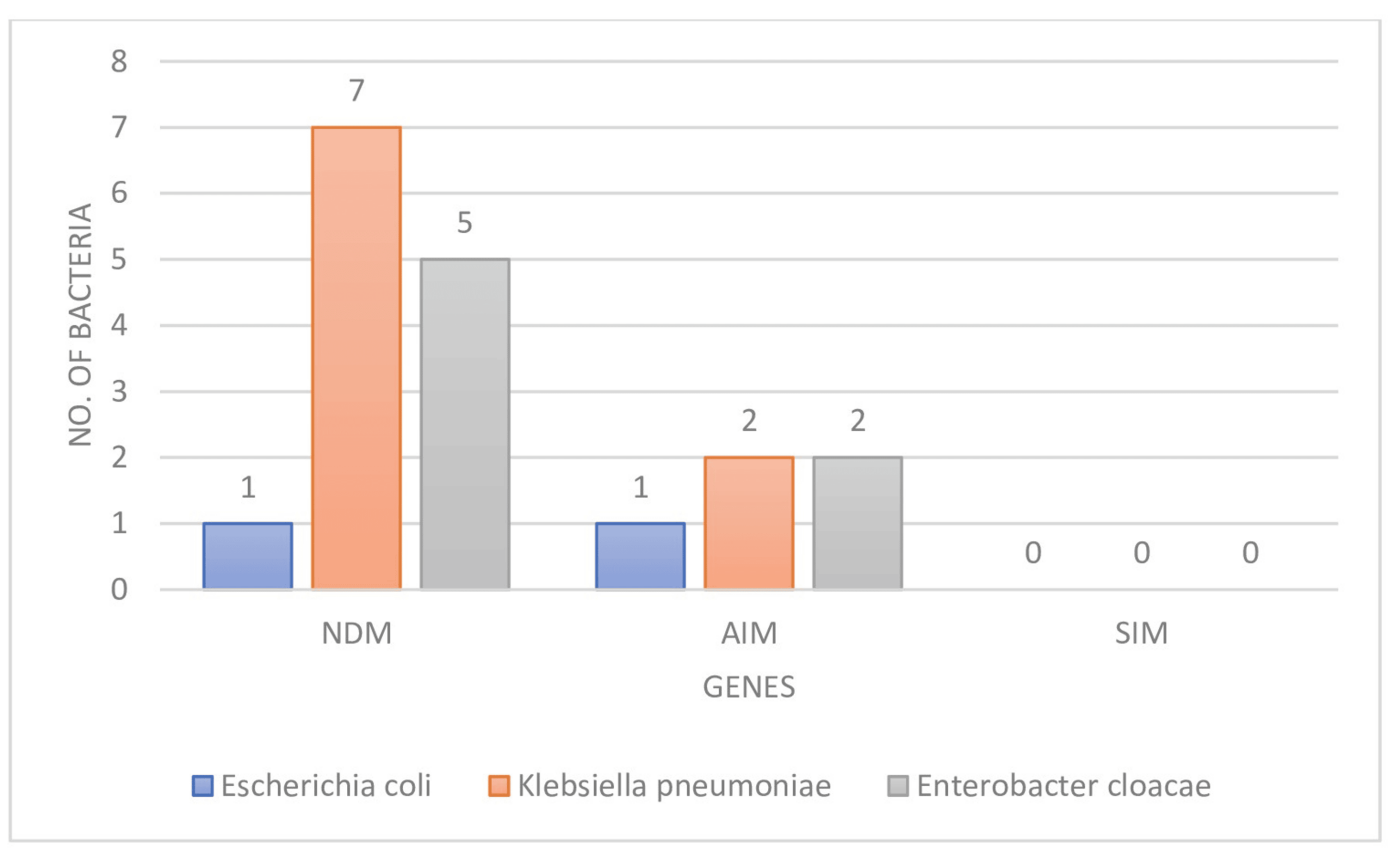
Numbers of bacteria with three carbapenem resistance genes isolated from pigeon feces in Jeddah, Saudi Arabia Metallo-β-lactamases genes: NDM=New Delhi metalo beta-lactamase, AIM=Adelaide imipenemase, SIM=Seoul imipenemase

The carbapenem resistance determining genes were present in 14/50 (28%) of the 50 Gram-negative antibiotic-resistance rods that were isolated from pigeon feces. The most common gene, found in 13 of 50 (26%) bacterial isolates, was NDM. It was present in 14% (7/50), 10% (5/50), and 2% (1/50) of *K. pneumoniae, E. cloacae*, and *E. coli* respectively. AIM beta-lactamase was found in five isolates out of 50 (10%); however, 4/50 (8%) isolates had both NDM and AIM genes, while SIM was not found in the examined isolated bacteria (Figures 2, 3). 

## Discussion

This study reports on a potential pathogen isolated from pigeon feces in Jeddah, Saudi Arabia. This finding demonstrates that the pigeons residing in various parks were carriers of *Enterobacter cloacae, Escherichia coli, *and* Klebsiella pneumoniae.* While there were no differences between the parks tested and the isolation and molecular recognition methodologies followed by previous studies [4-6], the determined prevalence depended on the samples collected; among other factors investigated, the sample size and area may vary. 

According to Tadesse DA et al. and Hur J et al., antibiotic drugs are essential for reducing the fatality and illness rate linked to communicable diseases in humans and animals. One of the most common animal contagious diseases treated with antibiotics is intestinal disease. Nevertheless, the extensive use of antibiotics in humans and animals has resulted in antibiotic-resistant bacteria [21,22]. While few antibiotics used in animal farming treat human infection and several animal pathogens are zoonotic, the development of resistant and multidrug-resistant pathogens among animal isolates is a medical issue [23]. Thus, we examined the sensitivity profile of *E. cloacae, E. coli, and K. pneumoniae* that were detected in pigeon feces in the current study. 

Urban pigeons may feed on trash or adjacent trash cans, which makes them susceptible to contamination by medically significant bacteria or by leftover antimicrobials and chemicals. When they interact with people in cities, they could also become contaminated [1,10]. Considering the location, it is difficult to pinpoint the roots of such pigeons in the city of Jeddah. Like any other major city, there are nosocomial areas, public parks, and plazas dispersed throughout Jeddah. Nonetheless, no open sewage networks or sewage plant treatments are located close to the city locations that were sampled, which may have contaminated the pigeons. 

Antimicrobial drugs provide selective pressure on microorganisms, which leads to the development of resistance determinants that can spread throughout different bacterial populations. According to our research, drug-resistant species of *E. coli, E. cloacae, *and *K. pneumoniae* have been recovered from pigeon feces, which may reflect the excessive usage of these chemicals in our community. 

Even though some authors have reported *E. coli* resistance to common bird medicine antibiotics groups like carbapenem, second-generation cephalosporin, third-generation cephalosporin, dihydrofolate reductase inhibitor/sulfonamide, penicillin-like antibiotics/beta-lactamase inhibitors, fluoroquinolone, and aminoglycosides, our findings have not been compared to any scientific publications in the literature [24,25]. 

It is noteworthy that the pigeons under investigation were not examined because of any disease, but rather because of the possibility that they harbor microorganisms that could be dangerous to people. Consequently, it is concerning to discover multiple antibiotic-resistance in infectious or commensal *E. coli, E. cloacae, *and* K. pneumoniae *from pigeons, especially if the genetic coding for these phenotypes can spread to other microbes or if people or other animals encounter these antibiotic-resistant bacteria. We did not investigate the possible factors that could be linked to the selective pressure that produced multiple resistances, but phenotypic evidence from other studies suggests that one of them could be co-selection [8,25,26]. 

Although there is inadequate information on antibiotic sensitivity patterns of human assumed pathogens detected from pigeons, previous regional studies displayed higher resistance percentages of *E. coli* isolated from infected humans to antimicrobials such as sulfamethoxazole-trimethoprim, quinolones, and cephalosporins [27,28]. Nonetheless, in this investigation, every isolate exhibited 100% resistance to meropenem, 90% resistance to cefuroxime, 56% resistance to gentamicin, and 52% resistance to amoxicillin/clavulanic acid. 

A higher percentage of ampicillin resistance (76%) was found in *E. coli* isolates of human origin that were in studies published between 1996 and 2007, considering other developing countries in other geographic locations, such as Asia and Africa. There have also been reports of emerging third-generation cephalosporin resistance (19%) and gentamycin resistance (13%) [4]. Other international data have indicated elevated levels of resistance in *E. coli* to quinolones such as nalidixic acid, ampicillin, trimethoprim, sulfamethoxazole, and the combination of sulfamethoxazole-trimethoprim [29]. 

Antimicrobial resistance has been noted for a few antibiotics crucial for human medication, particularly in exposed hosts. This behavior raises the prospect that *E. coli, E. cloacae, *and *K. pneumoniae* discovered from recent pigeon feces could act as a reservoir of antibiotic-resistant bacteria and/or resistance genes that could be transferred to pathogens through the environmental chain. 

NDM beta-lactamase was the most frequently found gene in this study (26%), followed by AIM beta-lactamase gene (5%). The results of Wang A, Hu C showed Extended-Spectrum β-Lactamase (ESBL)-producing *Escherichia coli *strains from pigeons carrying blaCTX-M-1G genes were in line with this study [30]. The strains of *K. pneumoniae, E. cloacae, *and* E.coli *in this investigation that carried these two-beta lactamase (NDM and AIM) genes were resistant to amoxicillin/clavulanic acid, cefuroxime, gentamicin, and meropenem. According to this data, there may be a chance for resistant *K. pneumoniae, E. cloacae, *and* E. coli* to spread between several hosts within the same area. Thus, it's essential to keep an eye on the AMR of *K. pneumoniae, E. cloacae, *and* E. coli* from pigeons to prevent the spread of resistance strains among various hosts in the same area and prevent their continued occurrence. 

AMR is a process that happens naturally and helps microorganisms survive. Nonetheless, this development has been influenced by human actions such as the overprescription of inappropriate drugs, the overuse of antibiotics as animal growth supplements, and the lack of new medicines [3]. In areas like ours with high rates of antimicrobial resistance, there is a need to watch these individuals more closely. It is advisable to schedule a follow-up for 72 hours following treatment in order to evaluate their therapeutic response [25]. 

According to this study, pigeon feces are a source of many zoonotic agents. Therefore, continuous surveys can evaluate the danger of human disease transmission from pigeons and contribute to its reduction.

Our study is still limited by the difficulty of utilizing the term "molecular characterization" for even a single class of antibiotics when employing PCR-based identification of resistant indicators in resistant isolates. Therefore, the potential of whole genome sequencing to investigate the likely origins and characteristics of resistant strains should be the focus of our future research project. 

## Conclusions

This study suggests that resistant *K. pneumoniae, E. cloacae, *and* E. coli *could spread to several hosts in the same location. Thus, it is crucial to keep an eye on the AMR of *E. coli, E. cloacae, *and *K. pneumoniae *from pigeons to stop the recurrence and spread of resistant strains among other hosts in the same area. Proper infection control measures and precautions should be taken immediately.
