# Correction: Antimicrobial Susceptibility and Molecular Identification of Antibiotic Resistance Enteric Bacteria Isolated From Pigeon Feces in the City of Jeddah, Saudi Arabia

**DOI:** 10.7759/cureus.c375

**Published:** 2025-11-11

**Authors:** Mohamed Elmutasim A Elsheikh, Maher Alandiyjany, Manal El Said, Faten Abouelmagd, Nadeem Ikram, Muhammad Awais, Elshiekh B Khidir, Wafaa M Abdulrahaman, Hassan Elsiddig Hag Elsafi, Omeima Salih

**Affiliations:** 1 Department of Microbiology, General Medicine Practice, Batterjee Medical College, Jeddah, SAU; 2 Department of Clinical Laboratory Sciences, Faculty of Applied Medical Sciences, Umm Al-Qura University, Makkah, SAU; 3 Department of Microbiology and Infection Prevention and Control Unit, Theodor Bilharz Research Institute, Giza, EGY; 4 Department of Medical Parasitology, Faculty of Medicine, Sohag University, Sohag, EGY; 5 Department Microbiology, Ahfad Centre for Science and Technology, Ahfad University for Women, Omdurman, SDN; 6 Department of Public Health, School of Health Sciences, Ahfad University for Women, Omdurman, SDN

The affiliation for Maher Alandiyjany has been corrected from ‘General Medicine Practice, Batterjee Medical College, Jeddah, SAU’ to ‘Department of Clinical Laboratory Sciences, Faculty of Applied Medical Sciences, Umm Al-Qura University, Makkah SAU’.

